# Recent Molecular Evolution of Human Metapneumovirus (HMPV): Subdivision of HMPV A2b Strains

**DOI:** 10.3390/microorganisms8091280

**Published:** 2020-08-21

**Authors:** Naganori Nao, Miwako Saikusa, Ko Sato, Tsuyoshi Sekizuka, Shuzo Usuku, Nobuko Tanaka, Hidekazu Nishimura, Makoto Takeda

**Affiliations:** 1 Department of Virology III, National Institute of Infectious Diseases, Musashimurayama 208-0011, Japan; mtakeda@nih.go.jp; 2 Yokohama City Institute of Public Health, Yokohama 236-0051, Japan; mi00-saikusa@city.yokohama.jp (M.S.); sh00-usuku@city.yokohama.jp (S.U.); no01-tanaka@city.yokohama.jp (N.T.); 3 Virus Research Center, Clinical Research Division, Sendai Medical Center, Sendai 983-8520, Japan; ko-sato@med.tohoku.ac.jp (K.S.); hide-nishimura@mte.biglobe.ne.jp (H.N.); 4 Pathogen Genomics Center, National Institute of Infectious Diseases, Shinjuku 162-8640, Japan; sekizuka@nih.go.jp

**Keywords:** human metapneumovirus, HMPV, molecular epidemiology, molecular evolution, subtyping, nucleotide duplication

## Abstract

Human metapneumovirus (HMPV) is a major etiological agent of acute respiratory infections in humans. HMPV has been circulating worldwide for more than six decades and is currently divided into five agreed-upon subtypes: A1, A2a, A2b, B1, and B2. Recently, the novel HMPV subtypes A2c, A2b1, and A2b2 have been proposed. However, the phylogenetic and evolutionary relationships between these recently proposed HMPV subtypes are unclear. Here, we report a genome-wide phylogenetic and evolutionary analysis of 161 HMPV strains, including unique HMPV subtype A2b strains with a 180- or 111-nucleotide duplication in the G gene (nt-dup). Our data demonstrate that the HMPV A2b subtype contains two distinct subtypes, A2b1 and A2b2, and that the HMPV subtypes A2c and A2b2 may be different names for the same subtype. HMPV A2b strains with a nt-dup also belong to subtype A2b2. Molecular evolutionary analyses indicate that subtypes A2b1 and A2b2 diverged from subtype A2b around a decade after the subtype A2 was divided into the subtypes A2a and A2b. These data support the A2b1 and A2b2 subtypes proposed in 2012 and are essential for the unified classification of HMPV subtype A2 strains, which is important for future HMPV surveillance and epidemiological studies.

## 1. Introduction

Human metapneumovirus (HMPV) was first discovered in 2001, but seroepidemiological studies indicate that HMPV has been a major etiological agent of acute respiratory infections (ARIs) in humans for more than six decades [1]. HMPV circulates worldwide; about half of all children are infected by HMPV before 2 years of age, and most children are infected before 5 years of age [1]. Unfortunately, individuals infected with HMPV usually do not develop lifelong immunity against this virus, and reinfection occurs frequently [1,2]. Although HMPV generally causes mild-to-moderate ARIs in the healthy adult population, it causes severe ARIs in aged adults and in patients with underlying diseases, such as diabetes and cardiopulmonary disease [3,4,5]. Fatal outbreaks of HMPV in a long-term care facility have been reported [6,7].

HMPV is a non-segmented, negative-stranded RNA virus belonging to the family *Pneumoviridae*. Its viral genome is approximately 13 kb in length and contains eight genes (N, P, M, F, M2, SH, G, and L). These genes encode a total of nine proteins, including three surface glycoproteins: F (fusion), SH (small hydrophobic), and G (glycol-) proteins. The F protein is an essential protein for viral adsorption and fusion to host cells, acting via RGD-bound integrins, such as α_v_β_1_ integrin, and glycosaminoglycans, such as heparan sulfate, and is a major target antigen for neutralizing antibodies [8,9,10]. The SH protein may suppress the innate immune response of host cells via suppressing NF-κB activation [11]. This protein also has properties consistent with those of a viroporin and can modulate viral fusogenic activity [12]. The G protein of some HMPV lineages also binds to glycosaminoglycans on the host cell surface and contributes to HMPV infection [13,14]. Additionally, the HMPV G protein associates with RIG-I, inhibits RIG-I-dependent gene transcription, and suppresses host innate immune responses [15].

HMPV is classified into two antigenically distinct groups, A and B [16]. The G protein is the most variable of the HMPV proteins, and mutations of the G protein accumulate predominantly in its extracellular domain [17]. Each viral group is further divided into two subgroups—A1 and A2 in group A, and B1 and B2 in group B—based mainly on variations in the G gene [16,18]. Furthermore, within the A2 subgroup, there are two phylogenetically distinct clades, A2a and A2b [19]. We recently reported unique HMPV A2b strains with a 180- or 111-nucleotide duplication in the G gene (HMPV A2b_180nt-dup_ or A2b_111nt-dup_ strains, respectively) [20,21]. In addition, other studies have recently described novel HMPV clades A2b2 and A2c [22,23]. The phylogenetic and evolutionary relationships between the HMPV A2b nt-dup strains, A2b2 strains, and A2c strains are still unclear. The present study conducted a genome-wide phylogenetic and evolutionary analysis of 161 HMPV strains to address this question.

## 2. Materials and Methods

### 2.1. Clinical Specimens and HMPV Isolation

Clinical specimens (throat swabs, nasal swabs, nasal secretions, and nasal aspirate fluids) were collected from patients suffering from ARIs in Yokohama city, Japan, as part of the National Epidemiological Surveillance of Infectious Diseases (NESID), which is conducted to comply with the Infectious Diseases Control Law in Japan. Collected clinical specimens were tested for 15 major respiratory viruses including HMPV by conducting a multiplex RT-PCR assay using the Seeplex^®^ RV15 OneStep ACE Detection kit (Seegene, Seoul, Korea). In the clinical specimens that tested positive for HMPV, the G genes were amplified via PCR and then sequenced as described previously [21]. Clinical specimens containing the HMPV A2b_111nt-dup_ strains were used for viral isolation.

TMPRSS2-expressing VeroE6 cells (VeroE6/TMPRSS2) were grown in DMEM supplemented with 10% fetal calf serum (FCS) and antibiotics at 37 °C in a 5% CO_2_ atmosphere [24]. VeroE6/TMPRSS2 cells were inoculated with the HMPV A2b_111nt-dup_ strains from clinical specimens, then incubated with DMEM supplemented with 5% FCS and antibiotics until a cytopathic effect (CPE) was observed.

### 2.2. Full-Genome Analysis of HMPV

Total RNA was extracted from the supernatant of HMPV-infected cells using TRIzol LS Reagent (Thermo Fisher Scientific, Waltham, MA, USA). The extracted viral RNA was reverse-transcribed and tagged with index adaptors using the NEBNext Ultra II RNA Library Prep Kit for Illumina (New England Biolabs, Ipswich, MA, USA) in accordance with the manufacturer’s instructions. The resulting cDNA libraries were verified using the MultiNA System (Shimadzu, Kyoto, Japan) and quantified using a Quantus Fluorometer (Promega, Madison, WI, USA). The indexed libraries were then pooled and sequenced (300-bp paired-end reads) using the MiSeq instrument (Illumina Inc., San Diego, CA, USA). After sequencing, the reads were subjected to de novo assembly using the IVA software with the default settings [25].

### 2.3. Maximum Likelihood Phylogenies

Multiple-sequence alignments were constructed with the MAFFT software (version 7.407) using the accuracy-oriented method (L-INS-i) [26]. A maximum likelihood phylogenetic analysis was performed using the IQ-TREE 2 software (version 2.0.3) [27]. The best-fit model for each analysis was determined using ModelFinder implemented in IQ-TREE 2 [28]. The statistical significance of each tree topology was tested with bootstrapping (1000 replicates).

### 2.4. Molecular Clock Phylogenies

Dated phylogenies were estimated with the BEAST2 software (version 2.6.2), which uses a Bayesian Markov chain Monte Carlo (MCMC) approach [29]. Multiple-sequence alignments were constructed with the MAFFT software (version 7.407) as described above, and the best substitution model for each dataset was determined using the jModelTest software (version 2.1.10) [30]. The most appropriate combination of molecular clock and coalescent models was estimated using a path-sampling analysis. MCMC chains were run for 100 million generations, with sampling parameters and trees every 3000 generations.

### 2.5. Ethics Statement

These analyses were performed as a part of the NESID in Japan, as stipulated under the Infectious Diseases Control Law. The principles of the Declaration of Helsinki were strictly followed. Before collecting the clinical specimens for this study, the physicians in each medical institution obtained informed consent from the patients or their guardians. The Ethics Committee of Yokohama City Institute of Public Health (Analysis of human metapneumovirus epidemic in Yokohama city, 1 Feb 2017) and the National Institute of Infectious Diseases (873, 12 Mar 2018) approved this study.

## 3. Results

### 3.1. Isolation of HMPV A2b_111nt-dup_ Strains and the Phylogenetic Relationship among HMPV Strains

**Figure 1:**
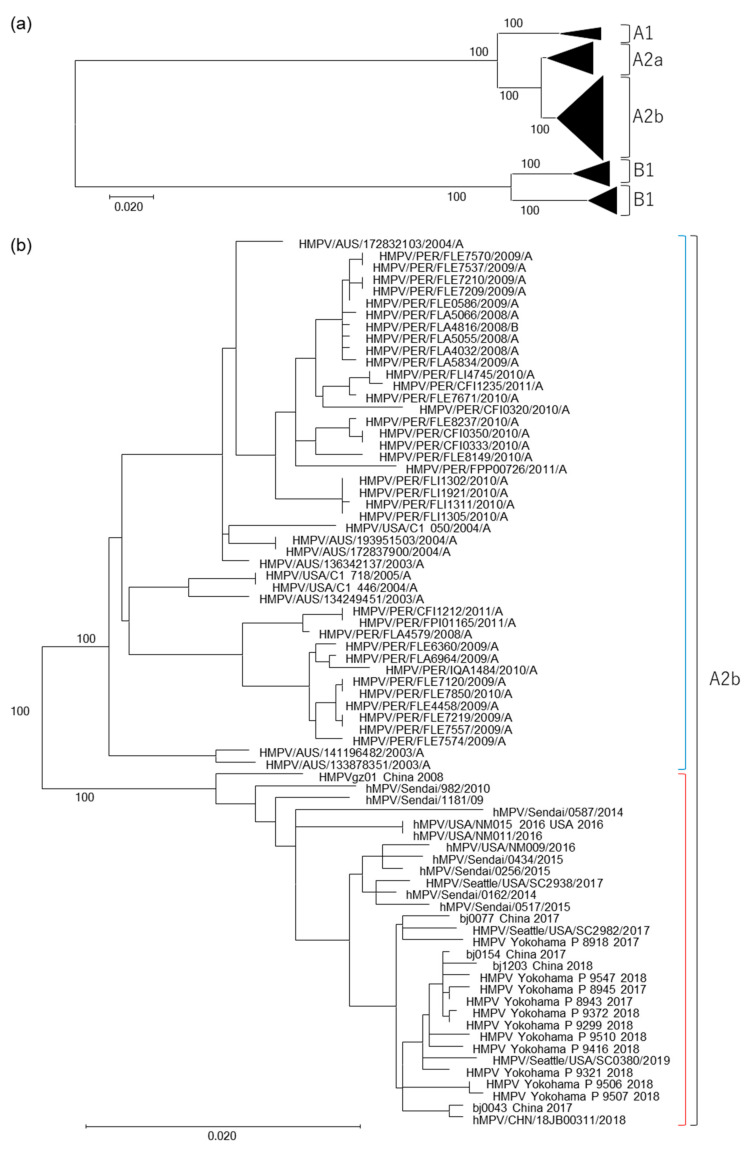
Maximum likelihood phylogenetic tree constructed based on HMPV full-genome sequences. (**a**,**b**) The tree was constructed using the maximum likelihood method with a GTR + F + R4 model and tested with bootstrapping (1000 replicates). Maximum likelihood trees constructed based on all the HMPV strains (**a**) and HMPV A2b strains (**b**) are shown. The HMPV A2b1 and A2b2 strains are shown as blue and red brackets, respectively.

Recently, epidemiological survey studies employing PCR assays have detected unique 180nt-dups and 111nt-dups in the G gene of HMPV subtype A2b strains [20,21,31]. Our previous work demonstrated that an HMPV A2b_111nt-dup_ strain is a predominant strain [32]. Here, we isolated 11 HMPV A2b_111nt-dup_ strains from clinical specimens and determined their full-genome sequences (Table 1, Dataset S1). In addition to these HMPV strains, all the HMPV strains with full-genome sequences available in the Virus Pathogen Resource database (http://www.viprbrc.org/brc/home.spg?decorator=vipr) (as of 12 March 2020), except for those whose listed sequences contained ambiguous nucleotide(s), were used in our phylogenetic analysis. The full-genome sequences of the HMPV strains isolated in Sendai city, Japan, including the HMPV A2b_180nt-dup_ strains, were also used in the analysis [24]. The results from this phylogenetic analysis of the HMPV full-genome sequences demonstrate that the HMPV A2b clade contains two distinct subtypes, A2b1 and A2b2, as previously proposed [23] (Figure 1). The HMPV A2b_111nt-dup_ strains isolated in Yokohama city belong to subtype A2b2 together with seven other HMPV strains that were isolated in Sendai city, Japan, four of which have a 180nt-dup and three of which have no nt-dup. Twelve HMPV strains isolated in China or the USA also belong to subtype A2b2. Of these 12 strains, five have a 111nt-dup, and the other seven strains have no nt-dup. Subtype A2b1 was found to contain 45 HMPV strains, isolated in Australia, Peru, or the USA. All 45 of the A2b1 strains have no nt-dup.

**Figure 2:**
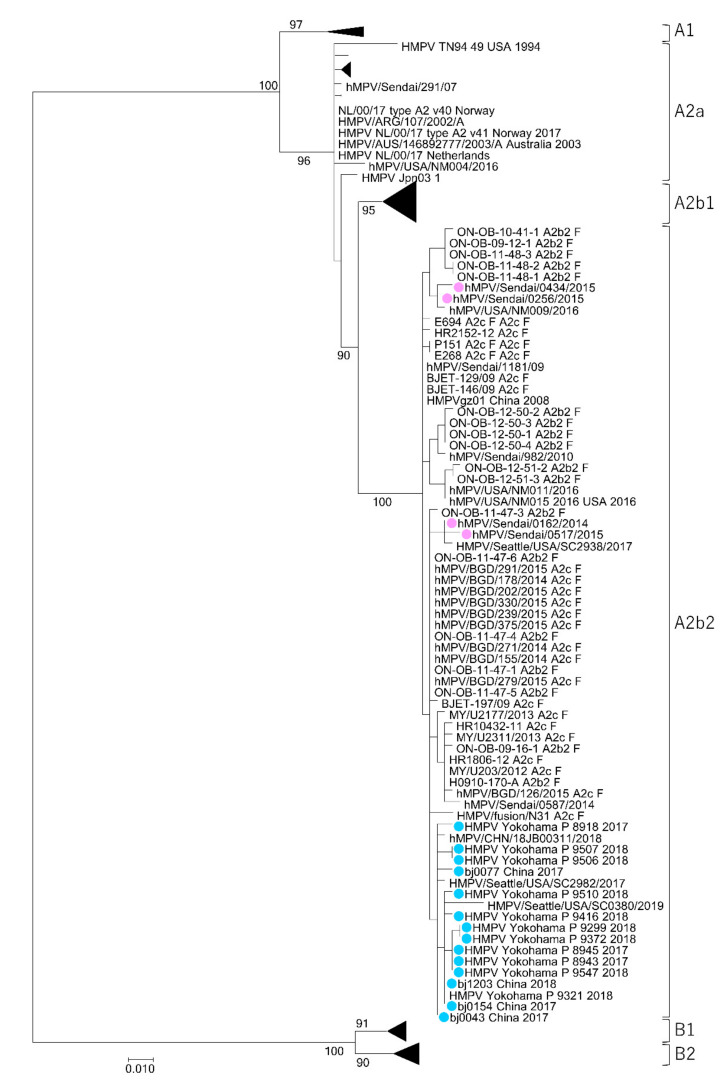
Maximum likelihood phylogenetic tree constructed based on a partial sequence of the HMPV F gene. The tree was constructed using the maximum likelihood method with a TIM2e + G4 model and tested with bootstrapping (1000 replicates). The HMPV A2b_180nt-dup_ and HMPV A2b_111nt-dup_ strains are shown as filled circles in pink and light blue, respectively.

**Figure 3:**
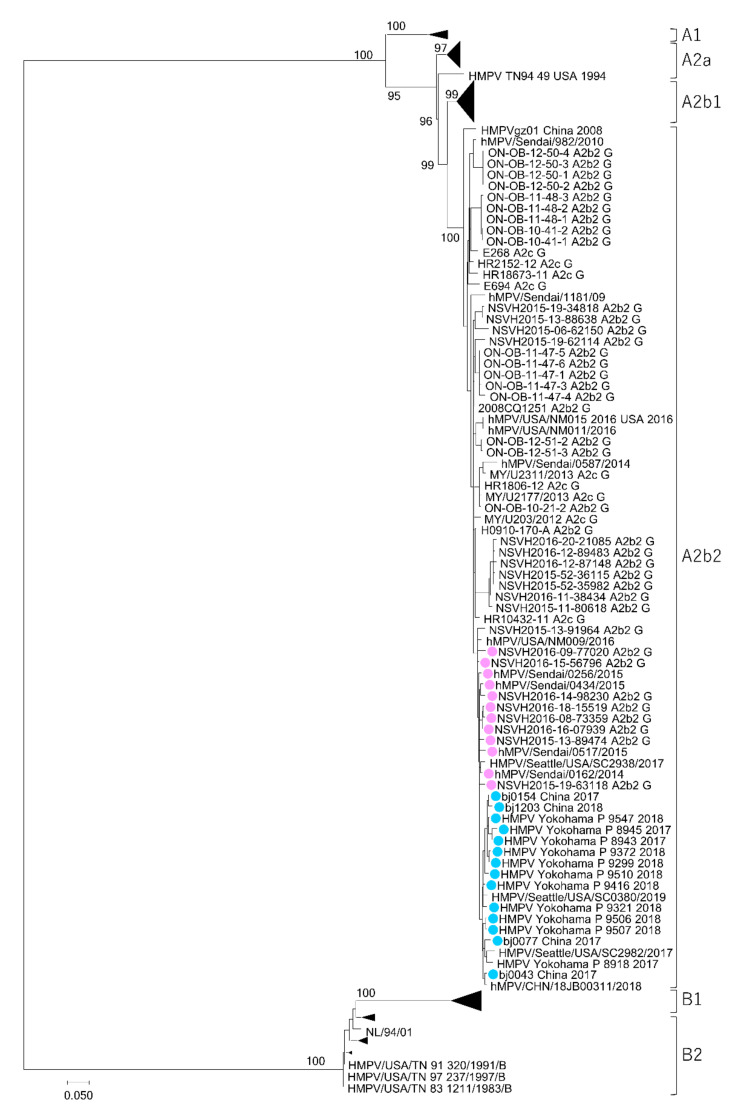
Maximum likelihood phylogenetic tree constructed based on a partial sequence of the HMPV G gene. The tree was constructed using the maximum likelihood method with a GTR + F + G4 model and tested with bootstrapping (1000 replicates). The HMPV A2b_180nt-dup_ and HMPV A2b_111nt-dup_ strains are shown as filled circles in pink and light blue, respectively.

In addition to the HMPV subtypes A2b1 and A2b2, another novel HMPV subtype A2c was recently reported [22]. To clarify the phylogenetic relationships among the HMPV strains, the genome sequences of the HMPV A2b2 and A2c strains from previous studies were obtained from the National Center for Biotechnology Information (NCBI) nucleotide sequence database (https://www.ncbi.nlm.nih.gov/) [22,23,31,33,34,35,36]. Unfortunately, no full-genome sequences for HMPV A2b2 and A2c were available, so the phylogenetic analysis was performed using partial sequences of the F and G genes. Although the resolution and bootstrap values of this phylogenetic analysis were not as high as those of the full-genome analysis, two distinct lineages for the HMPV A2b clade were also observed in the phylogenetic trees generated from the partial F and G gene sequences (Figure 2 and Figure 3). The HMPV A2b_180nt-dup_ and A2b_111nt-dup_ strains, HMPV A2b2 strains, and HMPV A2c strains were all found to belong to the same subtype. Notably, some of the HMPV A2b2 strains and HMPV A2c strains shared an identical nucleotide sequence in the analyzed F gene region (Figure 2).

### 3.2. Genetic Distances between HMPV Subtypes

The genetic *p* distances between individual subtypes and within each subtype were calculated based on the full-genome sequences of HMPV using the MEGA7 software (version 7.0.26) [37] (Table 2). The highest intra-subtype *p* distance (0.018) was found in the HMPV B1 subgroup. Notably, all the inter-subtype *p* distances, including the *p* distance between subtypes A2b1 and A2b2, were at least twice as high as the highest intra-subtype *p* distance (Table 2).

### 3.3. Evolutional Analysis of HMPV

**Figure 4:**
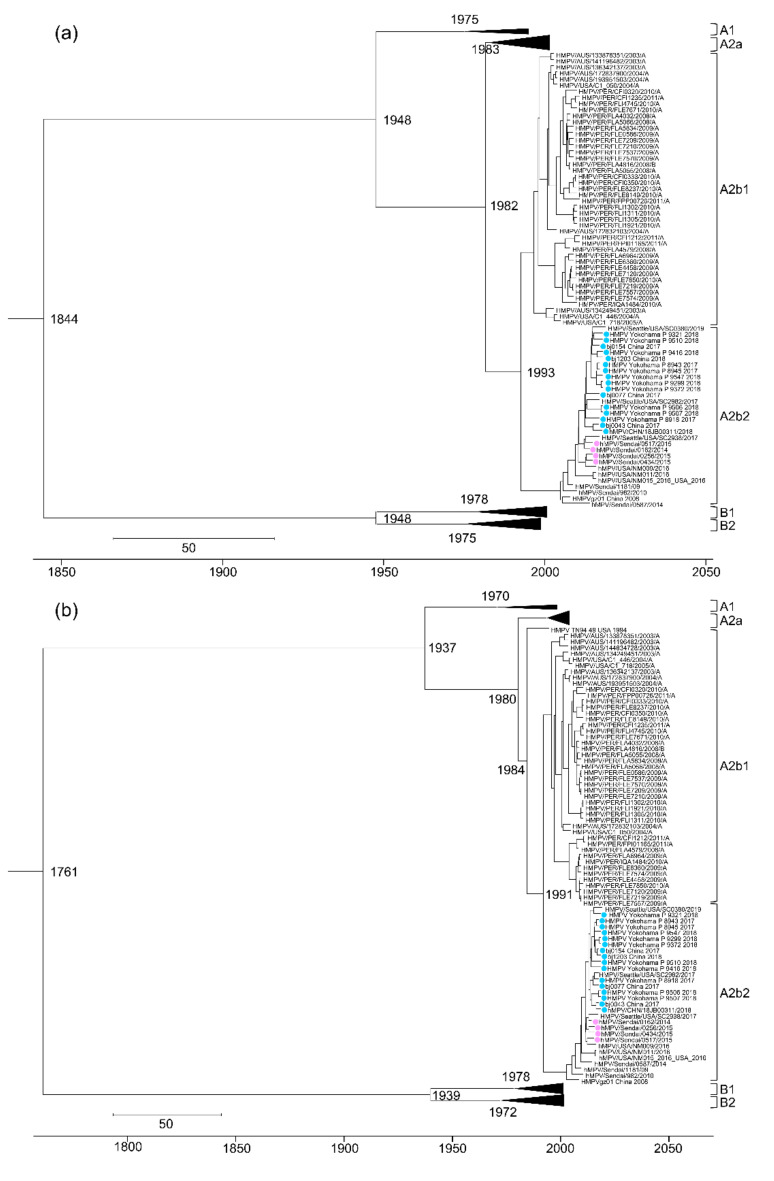
Markov chain Monte Carlo (MCMC) trees constructed based on the HMPV F and G genes. (**a**,**b**) MCMC trees constructed based on the HMPV F (**a**) and G genes (**b**) are shown. The MCMC tree based on the F gene was constructed using a TIM2 + I + G substitution model and an exponential relaxed molecular clock model. The MCMC tree based on the G gene was constructed using a GTR + I + G substitution model and an exponential relaxed molecular clock model. The HMPV A2b_180nt-dup_ and HMPV A2b_111nt-dup_ strains are shown as filled circles in pink and light blue, respectively. Scale bars indicate units in time (years). The full trees are shown in Figures S4 and S7.

In this study, we performed a molecular clock phylogeny analysis for all eight HMPV genes (Table 3). The MCMC trees generated from the sequences of the individual HMPV genes revealed the five known HMPV subtypes of A1, A2a, A2b, B1, and B2 (Figure 4, Figures S1–S8). As observed in the maximum likelihood tree generated based on HMPV full-genome sequences (Figure 1), two distinct lineages within the A2b subtype (A2b1 and A2b2) were observed in all eight MCMC trees generated based on individual HMPV genes (Figure 4, Figures S1–S8). The estimated time (year) of the most recent common ancestor (tMRCA) for HMPV (i.e., across all subtypes) was quite remote, regardless of the HMPV gene used for the analysis, with estimates ranging from 1645 to 1858 (N, 1827; P, 1775; M, 1812; F, 1844; M2, 1859; SH, 1645; G, 1761; and L, 1754). The estimated tMRCAs of the two main subtypes, HMPV A and HMPV B, were quite similar, ranging from 1925 to 1950 for subtype A and from 1924 to 1948 for subtype B. The tMRCAs of the HMPV subtypes A1, A2, B1, and B2 were more recent (less than about 50 years ago), except the tMRCA for the HMPV subtype B2 based on the P gene, which was estimated as 1950. The tMRCAs of the HMPV subtype A2a, HMPV subtype A2b, and HMPV subtype A2b1 were also similar to one another, ranging from 1989 to 1994, 1980 to 1996, and 1991 to 1998, respectively. In contrast, the tMRCA of the HMPV subtype A2b2 was more recent, with estimates ranging from 2002 to 2005 (Table 3).

## 4. Discussion

Shortly after the initial discovery of HMPV, four subtypes (A1, A2, B1, and B2) were proposed [16]. Subsequently, the HMPV subtype A2 was divided into two subtypes, A2a and A2b [19]. A consensus has been reached among researchers regarding these five HMPV subtypes. However, individual researchers have used different methods and gene regions or lengths when proposing new subtypes, such as A2c, A2b1, and A2b2 [22,23,31,33,34,35,36]. The “A2c” subtype was provisionally proposed based on analyses using only a short region (321 nucleotides in length) of the F gene and limited numbers of HMPV strains [22]. The “A2b1” and “A2b2” subtypes were also proposed based on analyses using a short region (111 nucleotides in length) of the F gene [23]. These provisional new subtypes of A2b strains were still classified as part of subtype A2b in some studies [32,38,39]. Such variations in genotype nomenclature among researchers may cause confusion regarding HMPV molecular epidemiology. Here, we demonstrated that the HMPV A2b2 strains and A2c strains all belong to the same subtype, together with the HMPV A2b_180nt-dup_ and A2b_111nt-dup_ strains, based on phylogenetic analyses conducted using partial regions of the F and G genes. Additionally, some of the HMPV A2b2 strains and A2c strains had identical nucleotide sequences in the analyzed region of the F gene. These data strongly suggest that the recently proposed HMPV subtypes A2c and A2b2 are separate descriptions indicating the same subtype. The genetic distance between the subtypes A2b1 and A2b2, which is twice as high as the highest intra-subtype *p* distance, indicates that A2b1 and A2b2 are distinct subtypes.

Both HMPV novel subtypes A2b2 and A2c were first proposed in 2012 based on phylogenetic analyses using a partial sequence of the HMPV F gene [22,23]. The phylogenetic trees of the two studies that proposed these HMPV subtypes differed from one another, especially in their subdivision of the subtype A2 strains. Nidaira et al. reported that strains belonging to subtype A2 were subdivided into three clusters: A2a, A2b, and A2c [22]. In contrast, Regev et al. found that subtype A2 was first divided into subtypes A2a and A2b, and then subtype A2b was further divided into subtypes A2b1 and A2b2 [23]. This difference in the constructed phylogenetic trees may reflect the differences in the viral strains and F gene regions used for the analyses. In the present study, we found that the HMPV A2 strains were first divided into A2a and A2b, and the A2b strains were then further divided into two distinct subtypes, A2b1 and A2b2. The presence of subtypes A2b1 and A2b2 within the subtype A2b was observed in the maximum likelihood tree constructed from the HMPV full-genome sequences as well as in all eight MCMC trees constructed based on the individual HMPV genes (Table 4). These data support the A2b1 and A2b2 subtypes proposed by Regev et al. in their phylogenetic tree.

In the present study, estimates for the tMRCA of all the HMPV strains ranged from 1645 to 1858, whereas those for the tMRCA of individual HMPV subtypes (A1, A2, B1, and B2) were around the 1970s and 1980s. These data are consistent with findings from a previous study using all eight HMPV genes [38]. The divergence time for the HMPV subtypes A2a and A2b was estimated as 1971–1985. In contrast, the estimated divergence time of the HMPV subtypes A2b1 and A2b2 was more recent (1989–1996). Compared with other subtypes, including A2a and A2b1, subtype A2b2 appeared to have the most recent tMRCA estimates (2002–2005). Our molecular evolutionary analyses indicate that subtypes A2b1 and A2b2 diverged from subtype A2b approximately a decade after subtype A2 became divided into subtypes A2a and A2b.

The recent subtype classification of HMPV A2 strains is not uniform among researchers. Here, we analyzed 161 full-genome sequences of HMPV strains, and the results show that the HMPV subtype A2b is divided into two subtypes that can appropriately be called A2b1 and A2b2. The HMPV A2b_180nt-dup_ and A2b_111nt-dup_ strains, which are now the predominant HMPV strains, were found to belong to the HMPV subtype A2b2; therefore, HMPV strains belonging to the subtype A2b2 are likely to be continuously detected for several years, if not longer [32]. Thus, a unified classification for the HMPV subtype A2 strains is important for future HMPV surveillance and epidemiological studies.

## 5. Conclusions

HMPV is currently divided into five agreed-upon subtypes: A1, A2a, A2b, B1, and B2. In 2012, the novel HMPV subtypes A2c, A2b1, and A2b2 were proposed based on phylogenetic analyses using a short region of the F gene and limited numbers of HMPV strains [22,23]. Recently, individual researchers have used different methods and gene regions or lengths when describing new subtypes, such as A2c, A2b1, and A2b2 [31,33,34,35,36]. However, a genome-wide phylogenetic and evolutionary analysis of recently proposed novel HMPV subtypes (A2c, A2b1, and A2b2) has not yet been performed, and detailed phylogenetic and evolutionary relationships between these subtypes are still unclear.

The present study conducted a genome-wide phylogenetic and evolutionary analysis of 161 HMPV strains and demonstrated that HMPV A2b strains were divided into two distinct subtypes, A2b1 and A2b2, as proposed by Regev et al. in 2012 [23]. In addition, our data also demonstrated that the HMPV subtypes A2b2 and A2c are separate descriptions indicating the same subtype. These findings are essential for the unified classification of HMPV subtype A2 strains, which is important for future HMPV surveillance and epidemiological studies.

## Figures and Tables

**Table 1:** Human metapneumovirus (HMPV) strains isolated in this study.

Strain Name	Isolation Year	Isolation Cell	111nt-dup
HMPV/Yokohama/P8918/2017	2017	VeroE6/TMPRSS2	+
HMPV/Yokohama/P8943/2017	2017	VeroE6/TMPRSS2	+
HMPV/Yokohama/P8945/2017	2017	VeroE6/TMPRSS2	+
HMPV/Yokohama/P9299/2018	2018	VeroE6/TMPRSS2	+
HMPV/Yokohama/P9321/2018	2018	VeroE6/TMPRSS2	+
HMPV/Yokohama/P9372/2018	2018	VeroE6/TMPRSS2	+
HMPV/Yokohama/P9416/2018	2018	VeroE6/TMPRSS2	+
HMPV/Yokohama/P9506/2018	2018	VeroE6/TMPRSS2	+
HMPV/Yokohama/P9507/2018	2018	VeroE6/TMPRSS2	+
HMPV/Yokohama/P9510/2018	2018	VeroE6/TMPRSS2	+
HMPV/Yokohama/P9547/2018	2018	VeroE6/TMPRSS2	+

111nt-dup: 111-nucleotide duplications in the G gene.

**Table 2:** Genetic distances between and within HMPV subtypes based on analyses conducted using full-genome sequences.

-	Genetic Distance (*p*-Value) *			-	-	-
Subtype	A1	A2a	A2b1	A2b2	B1	B2
A1	0.017					
A2a	0.076	0.013				
A2b1	0.077	0.043	0.016			
A2b2	0.079	0.047	0.036	0.011		
B1	0.225	0.225	0.226	0.224	0.018	
B2	0.226	0.225	0.227	0.225	0.073	0.017

* Pairwise distances were calculated between individual genotypes, as well as within each genotype, using the MEGA7 software.

**Table 3:** Estimated time of the most recent common ancestor (tMRCA) for each HMPV subtype.

Subtypes	tMRCAs of Each Viral Gene							
	N	P	M	F	M2	SH	G	L
All	1827	1775	1812	1844	1859	1645	1761	1754
A	1938	1937	1943	1948	1950	1937	1937	1925
A1	1978	1972	1979	1975	1979	1972	1970	1973
A2	1977	1978	1980	1982	1985	1971	1980	1977
A2a	1994	1993	1993	1994	1994	1989	1993	1992
A2b	1992	1991	1992	1993	1996	1980	1991	1989
A2b1	1994	1994	1996	1997	1998	1991	1996	1995
A2b2	2003	2003	2004	2005	2005	2004	2002	2002
B	1945	1924	1940	1948	1948	1934	1939	1933
B1	1979	1984	1979	1975	1982	1969	1978	1980
B2	1978	1950	1972	1978	1975	1969	1972	1969

**Table 4:** Comparison between previous studies and the current study.

-	The Current Study	Regev et al.	Nidaira et al.
Reference	-	[23]	[22]
The number of viruses analyzed	161 strains	26 strains	41 strains
Analyzed region	Full genome sequences (13 kb)	Partial F gene sequences (111 b)	Partial F gene sequences (321 b)
Phylogenetic analysis	Maximum likelihood analysis (IQ-TREE 2)	Nearest neighbor joint analysis (Clustal X)	Neighbor-joining analysis (MEGA)
Molecular clock phylogenies	Bayesian MCMC approach (BEAST2)	ND *	ND
Classification of HMPV A2 strains	A2a, A2b1, and A2b2	A2a, A2b1, and A2b2	A2a, A2b, and A2c

* ND: Not done.

## Supplementary Materials

The following are available online at https://www.mdpi.com/2076-2607/8/9/1280/s1. Figure S1: MCMC tree constructed based on the HMPV N gene; Figure S2: MCMC tree constructed based on the HMPV P gene; Figure S3: MCMC tree constructed based on the HMPV M gene; Figure S4: MCMC tree constructed based on the HMPV F gene; Figure S5: MCMC tree constructed based on the HMPV M2 gene; Figure S6: MCMC tree constructed based on the HMPV SH gene; Figure S7: MCMC tree constructed based on the HMPV G gene; Figure S8: MCMC tree constructed based on the HMPV L gene; Dataset S1: Full-genome sequences of the HMPV strains determined in this study.
